# Facial cooling while using respiratory protective devices in the cold

**DOI:** 10.1093/annweh/wxag063

**Published:** 2026-09-02

**Authors:** Jenni Kaisto, Kirsi Jussila, Arto Reiman, Sirkka Rissanen

**Affiliations:** Comprehensive Security in Working Life, Finnish Institute of Occupational Health, Työterveyslaitos, Oulu 90032, Finland; Industrial Engineering and Management, University of Oulu, P.O. Box 8000, Oulu 90014, Finland; Comprehensive Security in Working Life, Finnish Institute of Occupational Health, Työterveyslaitos, Oulu 90032, Finland; Industrial Engineering and Management, University of Oulu, P.O. Box 8000, Oulu 90014, Finland; Industrial Engineering and Management, University of Oulu, P.O. Box 8000, Oulu 90014, Finland; Occupational Safety, Finnish Institute of Occupational Health, Työterveyslaitos 90032, Finland

**Keywords:** face, cooling, skin temperature, respirator, cold, FFR, PAPR

## Abstract

The use of respiratory protective devices (RPDs) is essential in various work environments, including those with dust generation. When the work environment is cold, the low ambient temperature can accentuate discomfort from cooling and may influence workers’ selection of RPDs, potentially affecting protective efficiency. This study aimed to quantify facial cooling while wearing particle-filtering facepiece respirators in cold conditions. Six different RPDs were selected: two filtering facepiece respirators (FFRs), two powered air-purifying respirators with constant airflow (PAPR_C_) and two breath-responsive air-supplied respirators (PAPR_BR_). Ten volunteers (six males and four females) participated, using the RPDs during rest and exercise for 40 min at an ambient temperature of −20 °C. Skin temperatures on four areas of the face and the air temperature inside the mask were recorded throughout the test. In addition, accumulated moisture in the facepiece was measured. The mean face skin temperature (MFST) was calculated as the average of the four skin temperatures. The results showed that MFST declined, on average, to 12.9 °C, 17.7 °C and 21.8 °C when PAPR_C_, PAPR_BR_ and FFR were used, respectively. In conclusion, the use of RPDs in cold conditions presents particular occupational health and safety risks. Moisture retention is high when RPDs are used in cold conditions, and accumulated moisture inside the facepiece may cause discomfort and impair visibility. PAPR_C_ decreases facial skin temperatures to an uncomfortable level, while PAPR_BR_ and FFRs reduce cooling in cold environments. However, the required level of protection should be prioritised, even in cold conditions.

What's Important About this Paper? This study investigated facial cooling while using respiratory protective devices in cold conditions. The results showed that use of powered air-purifying respirators with continuous airflow at −20 °C causes excessive facial cooling and discomfort for the user. While selecting respiratory protective devices for use, the level of protection needed should always be prioritised but this study indicates that working temperature should also be taken into consideration.

## Introduction

The use of respiratory protective devices (RPDs) is necessary outdoors when ambient breathing air contains airborne contaminants, fumes, vapours, gases, dust, pathogens, or particles that are potentially hazardous. In northern countries, RPDs are frequently used in cold outdoor work environments, for example, in the construction sector, mining industry and industrial maintenance work. Work tasks in such industries are often characterised by physical activities conducted in outdoor environments with varying weather and environmental conditions, introducing additional complexity to occupational health and safety (OHS) management (Risikko et al. 2003; Anttonen et al. 2009). Ensuring OHS and employee well-being in such contexts is not merely a regulatory requirement but a fundamental component of organisational social responsibility. Employers are, therefore, obliged to integrate these considerations into their operations. Measures to safeguard employees, such as providing appropriate protective equipment, training and support, should be regarded as a key objective for all socially responsible organisations (Jain et al. 2024).

RPDs can be categorised as half- or full-face filtering facepiece respirators (FFRs), tight- or loose-fitting powered-air-purifying respirators (PAPRs) and self-contained breathing apparatus. Unpowered FFRs impose an excess respiratory load on the wearer due to elevated breathing resistance and increased dead space. The mask increases dead space by promoting CO_2_ rebreathing—which, in turn, increases ventilatory demand (Harber and Beckett 2023). Temperature and humidity within the mask dead space can increase to uncomfortable levels and reduce the effectiveness of respiratory heat exchange (Roberge et al. 2012). FFR wearers must breathe surrounding air through the filters using their respiratory muscles. In contrast, PAPRs offer the advantage of high airflow supplied from the blower unit through filters, which eases breathing inside the facepiece. PAPRs are generally perceived as more comfortable and allow the wearer greater capacity to work (Johnson et al. 2005; Anil et al. 2023). Moreover, loose-fitting PAPRs with integrated helmets or hoods do not require a tight seal against the skin, making them suitable for most users regardless of facial shape or the presence of facial hair. However, wearing tight-fitting half- or full-face PAPRs may have either minimal (Zimmerman et al. 1991) or significant negative effects on cognitive performance (AlGhamri et al. 2013). Unlike PAPRs with continuous airflow, breath-responsive PAPRs are tight-fitting, air-filtering, fan-assisted masks in which air flows through the filters only during inhalation.

Numerous studies have reported that the use of RPDs increases the risk of heat strain in warm and hot work environments (Nielsen et al. 1987; DuBois et al. 1990; Laird et al. 2002). However, the effects of RPD use in cold environments comparable to northern winter conditions have been far less studied and mainly documented in conference proceedings (see Jensen et al. 2014; Rissanen et al. 2016). In cold work environments, the unprotected face is exposed to the surrounding atmosphere. Early research on unpowered protective full-face masks (M17) suggested negligible thermal effects on the wearer at −32 °C (Johnson et al. 1992). More recent evidence indicates that this assumption may not hold for modern devices. Studies using a Newton thermal manikin have shown that PAPRs decrease facial skin temperatures and cause facial discomfort even in cool conditions (10 °C) (Del Ferraro et al. 2018, 2020). Studies with human test participants in cold environments (−10 to −30 °C) demonstrate that continuous airflow can reduce facial skin temperatures to uncomfortable levels within 5 to 10 min (Rissanen et al. 2016). In addition, user comfort is generally poor due to cold air blowing onto unprotected facial skin. Cold inhaled air has been reported as uncomfortable at 5 °C and unbearable at −10 °C when using PAPRs in cold conditions (Jensen et al. 2014). In Arctic conditions, respiratory protection presents also significant practical challenges (Rintamäki et al. 2015; Johnson 2016). Recently, Peyre-Costa et al. (2024) reported that Arctic miners experienced difficulties using RPDs in cold environments, including fogging, wetness and reduced visibility. These issues occasionally led workers to remove their respirators during work.

The use of RPDs, particularly PAPRs, in cold environments may also pose several potential health-related impacts. Wearing a PAPR in cold temperatures results in a constant flow of cold air directly onto the face, causing pronounced face cooling and discomfort (Rissanen et al. 2016). Cold air flow on the face may induce an increase in blood pressure and reduce respiratory function (Gavhed et al. 2000). Facial cooling also triggers reflex vasoconstriction in the fingers and toes (Brown et al. 2003) and stimulates the parasympathetic pathway, leading to bradycardia (Hilz et al. 1999). Cooling facial skin to 13 to 15 °C was found to trigger bronchoconstriction at an ambient temperature of −17 °C, regardless of whether the inhaled air was cold or warm (Koskela and Tukiainen 1995). Furthermore, hyperventilation can exaggerate bronchoconstriction in individuals with asthma (Zeitoun et al. 2004).

The thermal effects of RPD use in cold environments have been recognised and studied in terms of thermal sensations (Jensen et al. 2014); however, changes in facial skin temperature during RPD use under cold conditions remain largely unexplored in the peer-reviewed literature. Existing studies are limited in both scope and depth (Jensen et al. 2014; Rissanen et al. 2016), indicating a notable gap in empirical research involving human participants. Most PAPRs are recommended by manufacturers for use at ambient temperatures no lower than −10 °C. However, in northern countries, the need to use PAPRs frequently arises at lower temperatures. For instance, in Finland, where this study was conducted, average winter temperatures in 2024 varied regionally from −1 °C in the southwestern area to −14 °C in eastern and northern Lapland, with the lowest daily temperatures reaching −44 °C in Lapland (Finnish Meteorological Institute c2025). In this study, RPD use was investigated at an ambient temperature of −20 °C. The purpose of this study was to quantify facial skin cooling associated with different types of RPDs (powered and nonpowered) during simulated outdoor work at −20 °C and to evaluate moisture accumulation within the respirators. Based on earlier knowledge on the practical problems associated with RPD use in the cold (Rintamäki et al. 2015; Johnson 2016; Peyre-Costa et al. 2024), it was hypothesised that exhaled moisture could freeze within the facepiece of RPD at low temperatures. Therefore, this study also focused on this phenomenon, measuring accumulated moisture in the RPD facepiece during cold exposure and visually inspecting devices for freezing. Our study provides empirical evidence on the challenges associated with RPD use in cold environments and contributes knowledge to support employers in implementing practices that mitigate cold-related OHS risks during RPD use.

## Methods

### Participants

Ten healthy volunteers (four females and six males) participated in the study. This sample was considered representative of typical working-age individuals in industries relevant to this study (Statistics Finland c2023). Test participants were nonsmoking and none of them had experienced any cold injuries. The anthropometric measurements of the participants are shown in Table 1. Three facial dimensions were measured: length (bridge of nose to chin, Menton–sellion), facial width (bizygomatic diameter) and mouth width (EN 136 1998). These three dimensions, along with facial depth, are included in the European standard EN 136:1998 for leakage testing of RPDs and are recommended for reporting in test documentation. Facial depth was not measured in this study due to the absence of an appropriate measuring instrument.

**Table 1:** Characteristics and relevant anthropometric data of the participants (*N* = 10, mean [SD]).

Age (y)	Height (cm)	Weight (kg)	Face length (mm)	Face width (mm)	Mouth width (mm)
**27.6 (11.9)**	174.3 (11.8)	75.5 (21.6)	113.5 (7.3)	116.9 (5.0)	47.8 (2.5)

The study was conducted in accordance with the recommendations of the Declaration of Helsinki (1983). Ethical approval for the research was obtained from the Ethical Committee of the Finnish Institute of Occupational Health (ETR 10/2016). All participants were fully informed of the experimental procedures and familiarised with them in detail, and each provided written informed consent prior to the experiment. Participants were instructed to notify the researchers if they felt excessively cold or wished to discontinue the experiment for any reason.

### Respiratory protective devices

Measurements were conducted with six RPDs of different types. These included four PAPRs, either breath-responsive (two units) or with continuous air flow (two units), and two FFRs composed of different materials. These respirators were selected for the study based on those typically used in Finnish industrial workplaces where RPDs are employed in the cold, such as open-pit mines and the steel industry. Detailed information on the used RPDs is presented in Table 2. During the exposure, the lowest selectable airflow rate was used for PAPRs with continuous airflow. Multiple sizes of respirator facepieces were available, and researchers selected the appropriately fitting model together with each participant.

**Table 2:** Studied RPD types and their abbreviations and descriptions.

Type of RPD	Description	Figure	Operating temperature
FFRd	Disposable filtering half-face mask with an exhalation valve. EN 149 FFP3. Assigned protection factor 20.	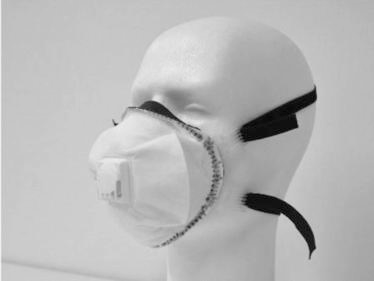	Not provided
FFRe	Reusable elastomeric half-face mask with an exhalation valve and integrated filter elements. EN 405 FFA1B1E1K1P3 RD. Assigned protection factor 10.	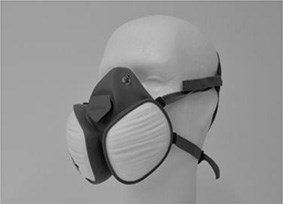	Not provided
PAPR_C_TF_	Tight-fitting full-face mask with an oronasal inner mask and a blower unit, continuous air flow (120 L/min) from side. EN 12942 TM3. Assigned protection factor 40.	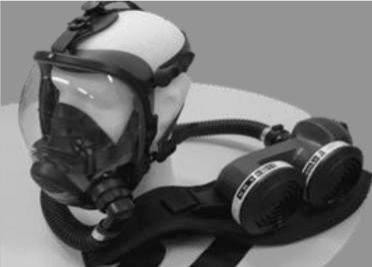	−10 to +30 °C
PAPR_C_LF_	Loose-fitting respirator integrated with a helmet and visor with a blower unit, continuous air flow (190, 205 and 220 L/min) from top of the helmet. EN 12941. Assigned protection factor 40.	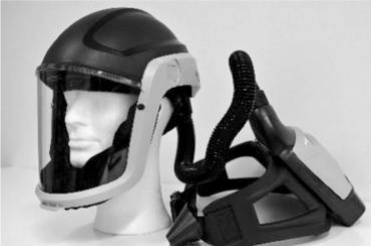	−10 to +55 °C
PAPR_BR_H_	Tight-fitting half-face mask with a blower unit, breath-responsive air flow (0 to 230 L/min) from side. EN 12942. Assigned protection factor 50.	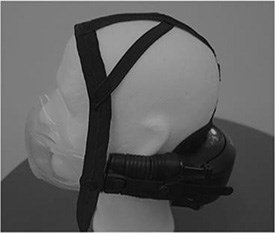	−10 to +45 °C
PAPR_BR_F_	Tight-fitting full-face mask with an oronasal inner mask and a blower unit, breath-responsive air flow (0 to 230 L/min) from side. EN 12942 Assigned protection factor 1,000.	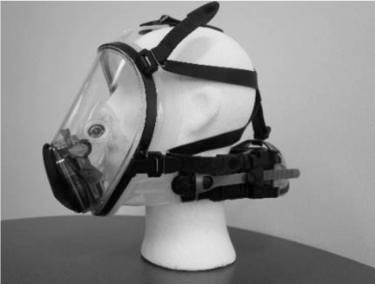	−10 to +45 °C

### Measurements of facial cooling

All participants completed six experimental sessions, each with a different RPD, in random order. Sessions were separated by 1 to 7 d. Skin temperatures were measured at four sites (forehead, cheek, nose, and chin) using thermistor sensors (2M21916, Dräger, Germany) and a data logger (SmartReader 7Plus ACR Systems, Canada), recording at 1 min intervals. Sensors on the cheek and forehead were positioned outside the half masks, and for full-face masks integrated with oronasal mask, these sensors were placed outside the inner mask. An additional temperature sensor was positioned inside the facepiece at the level of the cheekbone for PAPR_C_ and PAPR_BR_F_, near the mouth and nose for FFRs and PAPR_BR_H_, to measure the air temperature during exposure. The data logger was carried in the pocket of the participant's jacket.

The participants’ subjective thermal sensations (general and facial) and perceived exertion (RPE) were recorded using standardised scales (ISO 10551, 1995; Borg 1998) at the beginning of the test and after each phase of the protocol. The thermal sensation rating scale was as follows: −4 = very cold, −3 = cold, −2 = cool, −1 = slightly cool, 0 = neutral, 1 = slightly warm, 2 = warm, 3 = hot, 4 = very hot. The RPE scale ranged from 6 = no exertion at all to 20 = maximum exertion.

The participants wore clothing estimated to provide adequate protection for the climatic conditions (ISO 11079 2007): underwear (long-sleeved shirt and trousers), a middle layer (fleece jacket and trousers), a cold-protective clothing ensemble (jacket and trousers), safety shoes, winter gloves, headwear and a helmet. The use of RPDs was instructed and practised prior to the measurements, and correct donning of each device was supervised. Tests were conducted in a climate chamber at an ambient temperature of −20 °C, with air movement maintained below 0.3 m/s.

### Measurement of accumulated moisture in the facepiece of RPD

Accumulated moisture in the RPD facepiece during exposure was determined by measuring the weight of the facepiece before and after each session. Moisture accumulation was treated as a separate variable, not assumed to be directly linked to facial skin temperatures but used to explore the performance and usability of RPDs in the cold. It was hypothesised that moisture accumulating inside the mask could interfere with the proper functioning of the respirator, for example, by clogging the filters or valves. Therefore, the level of condensed exhaled moisture in the mask was measured to identify the threshold at which freezing and related functional disturbances might occur. Fogging and freezing of exhaled moisture in the facepieces were visually inspected throughout the exposure.

### Protocol

After instrumentation, the RPD was donned, and the power unit was switched on (except for FFRs), after which the participant entered the climate chamber. The test protocol lasted 40 min and comprised four consecutive phases: 15 min of standing still, 10 min of step exercise (step frequency 20 times/min, guided by a metronome; step height of 20 cm), 5 min of lifting a 3- or 5-kg dumbbell (women/men) from the ground to a table with the arm extended, then back down to the table and finally back to the ground and a final 10 min of standing in place. The protocol was designed to simulate light to moderately demanding outdoor work, incorporating both upper- and lower-limb activities. Work rate was estimated as light to moderate according to ISO 8996 (2004, Table A.1).

### Statistical analysis

Mean face skin temperature (MFST) was calculated as the average of the forehead, cheek, nose and chin temperatures. Two variables—minimum MFST during the exposure (MFST_min_) and MFST cooling rate during the first 15 min of the exposure (MFST_cr_)—were analysed using paired-samples *t*-tests across the six different respirator types. In addition, the minimum air temperature inside the mask during the exposure (min T_air_) was compared separately between the half- and full-face masks. The Mann–Whitney test was used to examine whether gender was associated with facial skin temperature, and Spearman's correlation test was used to assess the relationship between age and facial temperature. All analyses were performed using a statistical software package (IBM SPSS Statistics 30). Statistical significance was set at *P* < 0.05. All data are reported as mean (SD).

## Results

### Local skin temperatures

Minimum local skin temperatures were lowest when wearing PAPR_C_TF_ and PAPR_C_LF_ (Table 3). In one participant wearing PAPR_C_LF_, the cheek temperature dropped as low as 5 °C. Nose, chin and cheek skin temperatures were slightly lower for PAPR_C_LF_ compared with PAPR_C_TF_, with the difference in chin temperature reaching statistical significance. Use of PAPR_BR_F_ resulted in higher local skin temperatures compared with PAPR_C_TF_ and PAPR_C_LF_, with significant differences observed in the cheek, chin and forehead temperatures. Nose and chin temperatures did not remarkably drop when using FFRd and FFRe, as these areas were warmed both by the face itself and by exhaled air. Cheek and forehead skin temperatures are not covered by the half-face masks in FFRd, FFRe and PAPR_BR_H_ and were, therefore, exposed to the ambient temperature of −20 °C. A statistically significant difference between genders was observed for minimum chin temperatures while wearing PAPR_C_TF_, with males showing, on average, 3.4 °C lower temperatures than females (*U* = 1.00, *P* = 0.019). Age was not significantly correlated with local facial skin temperatures.

**Table 3:** Minimum local skin temperatures (°C) during 40-min exposure to −20 °C. Mean (SD). *n* = 10. Significance *P* < 0.05.

RPD	Nose	Chin	Cheek (a)	Forehead (a)
FFRd	25.8 (3.0)	26.8 (5.6)	18.8 (1.3)	21.8 (3.2)
FFRe	17.5 (3.4)^a^	21.0 (3.3)^a^	13.8 (2.2)^a^	22.5 (3.6)
PAPR_C_TF_	12.2 (4.2)^a^	11.9 (2.6)^a^	10.1 (2.4)^a^	15.6 (3.6)^a^
PAPR_C_LF_	11.0 (5.6)^a^	8.7 (4.3)^ab^	8.4 (3.8)^a^	18.8 (4.1)^a^
PAPR_BR_H_	12.8 (4.2)^a^	14.8 (4.9)^ac^	12.4 (3.3)^abc^	23.9 (3.5)^bc^
PAPR_BR_F_	14.2 (5.0)^a^	18.4 (6.3)^abc^	16.5 (2.8)^abc^	23.7 (4.2)^bc^

(a) Cheek and forehead are exposed to ambient temperature while wearing FFRd, FFRe, PAPR_BR_H_.

^a^ *P* < 0.05 compared with FFRd.

^b^ *P* < 0.05 compared with PAPR_C_TF_.

^c^ *P* < 0.05 compared with PAPR_C_LF_.

### Air temperature inside the masks

Air temperature inside the facepiece (minT_air_) decreased to −10.5 °C and −10.1 °C when using PAPR_C_TF_ and PAPR_C_LF_, respectively, at −20 °C (Table 4). The next lowest minT_air_ values were observed with PAPR_BR_H_ and PAPR_BR_F_, measuring 4.7 and −0.0 °C, respectively. The warmest minT_air_ was recorded with FFRd and FFRe; however, FFRe produced a minT_air_ that was 10 °C lower than that of FFRd (*P* < 0.001).

**Table 4:** Minimum MFST during exposure (MFST_min_, °C), cooling rate of MFST during the first 15 min of exposure (MFST_cr_, °C/min) and minimum air temperature inside the mask during exposure (minT_air_, °C) (*n* = 10, mean (SD)). Significance *P* < 0.05.

RPD	MFST_min_	MFST_cr_	minT_air_
FFRd	24.3 (1.3)	0.1 (0.3)	23.6 (2.9)
FFRe	19.3 (2.0)^a^	0.4 (0.1)^ac^	13.5 (4.0)^a^
PAPR_C_TF_	13.1 (1.6)^bc^	0.9 (0.1)^bc^	−10.5 (1.4)^bc^
PAPR_C_LF_	12.7 (2.7)^bc^	1.0 (0.2)^bc^	−10.1 (3.2)^bc^
PAPR_BR_H_	16.5 (2.8)^a^	0.6 (0.2)^ab^	4.7 (5.4)^ab^
PAPR_BR_F_	18.8 (3.0)	0.4 (0.2)	−0.0 (5.0)

^a^Compared with FFRd.

^b^Compared with PAPR_BR_F_.

^c^Compared with PAPR_BR_ H_.

### Mean facial skin temperature

**Figure 1:**
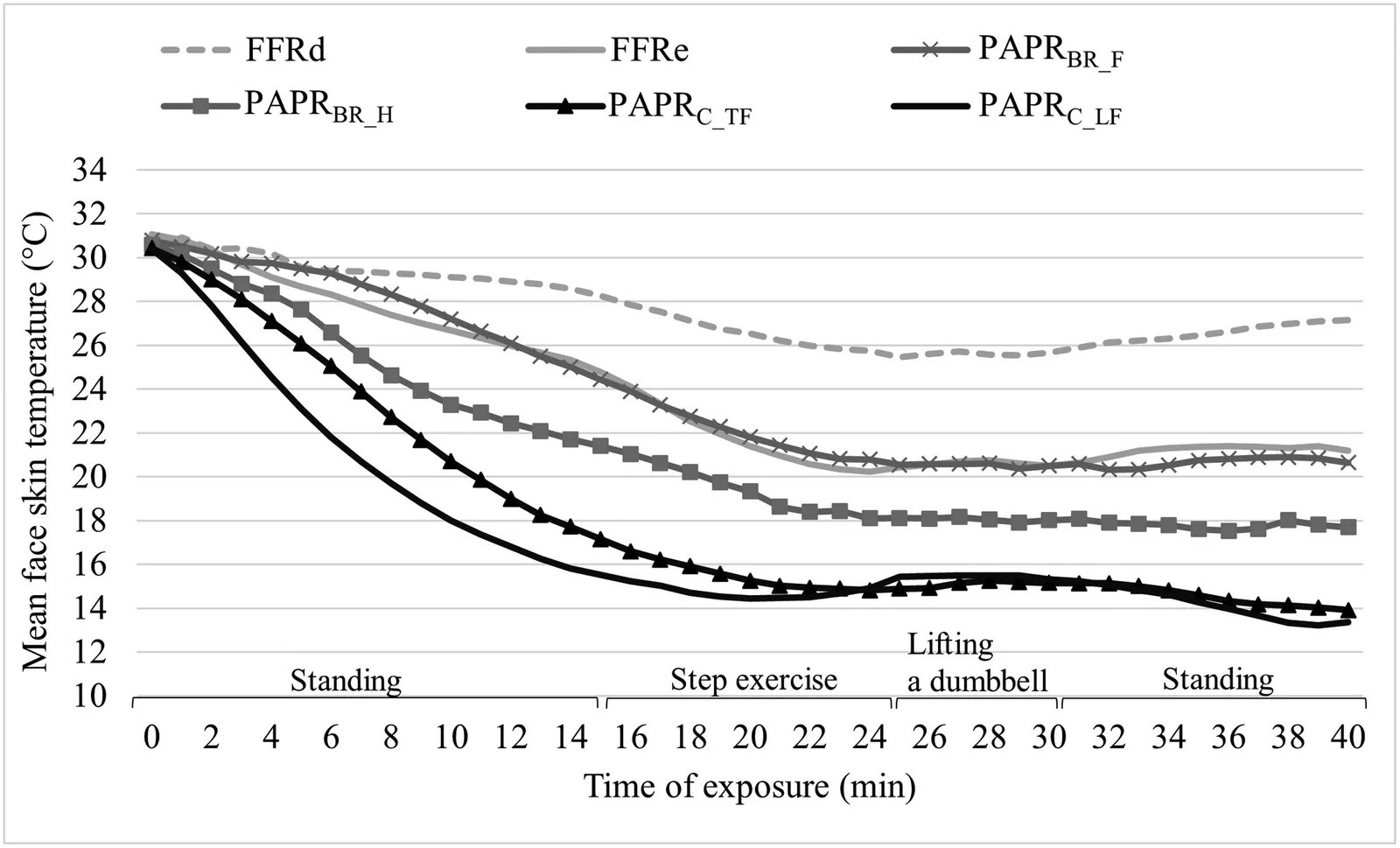
Mean facial skin temperature while wearing different types of RPDs at −20 °C during standing (0 to 15 and 30 to 40 min) and exercise periods. Values are means (*n* = 10).

Wearing FFRd resulted in the highest MFST and lowest cooling rate (Fig. 1). FFRe and PAPR_BR_F_ exhibited similar cooling patterns. Facial cooling was most rapid and pronounced when wearing PAPR_C_LF_ or PAPR_C_TF_ during the first standing period (0 to 15 min). During the exercise phases (15 to 30 min), the rate of cooling levelled off. In the second rest period (30 to 40 min), MFST either began to decrease (PAPR_C_LF_ and PAPR_C_TF_), slightly increased (FFRd, FFRe and PAPR_BR_F_) or remained stable (PAPR_BR_H_).

Table 4 shows detailed differences in MFST and minimum air temperatures inside the mask (minT_air_) between the different RPD types. The coldest MFST was observed while wearing PAPR_C_LF_ and PAPR_C_TF_, compared with other RPDs. Similarly, the cooling rate (MFST_cr_) was the highest for these respirators. No significant differences in MFSTs were detected between PAPR_C_LF_ and PAPR_C_TF_. FFRd produced MFST_min_ values that were 5.0 °C and 7.8 °C higher (*P* < 0.001) than those of the other half masks, FFRe and PAPR_BR_H_, respectively. When wearing PAPR_BR_H_ or PAPR_BR_F_, the MFST cooling rate was slower (*P* < 0.001) and MFST_min_ was higher (*P* < 0.001) compared with PAPR_C_LF_ and PAPR_C_TF_. Only the MFST cooling rate of PAPR_BR_H_ was higher than that of PAPR_BR_F_, while MFST_min_ did not differ significantly. No statistical differences in MFST_min_ were found between genders; however, a significant difference in MFST_cr_ was observed while using PAPR_BR_H_, with males exhibiting a significantly lower cooling rate than females (0.21 °C/min, *U* = 1.00, *P* = 0.019). Age was not significantly correlated with mean facial skin temperatures.

### Thermal sensations and perceived exertion

The participants rated both exercise tasks as involving light exertion, with no differences in RPE observed between the different RPDs.

Thermal sensation decreased with prolonged exposure to the cold. Sensations increased slightly during the work tasks but remained below thermoneutral conditions (slightly cool to cool). The coldest sensations were reported while wearing PAPR_C_TF_ and PAPR_C_LF_. Breath-responsive devices were perceived as less cold than continuous-flow PAPRs but colder than FFRd and FFRe. Perception of the coldest areas of the face varied with the type of respirator. While wearing FFRs, the cheeks and skin beneath the edge of the respirator were most frequently reported as the coldest. The chin was most often perceived as the coldest when using full-face masks. Eye wetting and nasal congestion were commonly reported with PAPRs.

### Moisture

Accumulated moisture in the facepiece of RPDs during exposure is presented in Table 5. The greatest amount of moisture was observed in PAPR_C_LF_, in which also substantial fogging and freezing of exhaled moisture on the surface of the visor occurred. Moisture accumulation in PAPR_C_LF_ was significantly higher than that in PAPR_C_TF_ and PAPR_BR_F_ (*P* < 0.05). Fogging and freezing of exhaled moisture were visually evaluated as slightly less severe in PAPR_C_TF_ and PAPR_BR_F_. A substantial amount of moisture was also measured in FFRd. Some of this moisture froze on the mask material. Additionally, moisture accumulation in PAPR_BR_F_ was significantly greater than in PAPR_BR_H_ (*P* < 0.001) and PAPR_C_TF_ (*P* < 0.05). No freezing of moisture was observed that would impair the normal function of the facepiece, such as clogging filters or valves.

**Table 5:** Accumulated moisture in the facepieces of the studied RPDs during 40-min exposure (*n* = 10, mean (SD)). Significance *P* < 0.05.

RPD	Moisture in the facepiece of RPD (g)
FFRd	5.7 (2.3)
FFRe	3.6 (0.9)^a^
PAPR_C_TF_	3.0 (1.2)
PAPR_C_LF_	6.4 (3.0)^bc^
PAPR_BR_H_	2.7 (0.7)
PAPR_BR_F_	4.1 (1.0)^bd^

^a^Compared with FFRd.

^b^Compared with PAPR_C_TF_.

^c^Compared with PAPR_BR_F_.

^d^Compared with PAPR_BR_H_.

No functional issues were observed with the RPDs tested, despite the study being conducted outside their specified operating range.

## Discussion

The use of RPDs is generally known to increase psychophysiological strain at work by increasing respiratory resistance, adding weight and hindering communication. Experiences from the outdoor workers indicate that low temperatures create additional challenges such as fogging and icing of visors and freezing of exhaled moisture. In particular, PAPRs have been criticised for excessively cooling the face. The use of RPDs represents an OHS concern that requires careful management and coordinated responsibility to ensure effective protection, particularly in cold environments where discomfort or usability issues may lead to situations in which the device is not worn at all.

According to the literature, cooling of the skin to 15 °C produces sensations of cold and pain (Park and Kim 2013), often perceived as burning, aching or pricking. Further cooling below 10 °C eventually leads to sensory numbness, which is usually the first sign of frostbite (Castellani et al. 2006). In this context, the results of the present study indicate that facial cooling is excessive when PAPRs are used in cold environments. Continuous air flow during 40 min of cold exposure lowered facial skin temperatures below 15 °C, with one participant experiencing a local skin temperature as low as 5 °C. While wearing breath-responsive PAPRs, where air flows into the mask only during inhalation, the MFST decreased to approximately 17 °C (PAPR_BR_H_) and 19 °C (PAPR_BR_F_). While using disposable FFRd, the MFST remained above 24 °C, whereas elastomeric FFRe produced a mean skin temperature approximately 5 °C lower. Subjective thermal sensation ratings reflected these differences, being closely influenced by the degree of facial cooling associated with each type of RPD.

The use of tight- or loose-fitting PAPR_C_ resulted in significantly faster and more pronounced cooling of facial skin temperatures. Due to the continuous flow of cold ambient air, the minT_air_ inside the mask reached −10 °C during exposure to −20 °C. Furthermore, in these models, the breathing tube (60 to 80 cm long) connecting the helmet to the blower unit remains exposed to the ambient temperature.

When using breath-responsive PAPR_BR_, cold air is delivered into the mask only during inhalation. The breathing tube in these models is short (∼20 cm) because the blower unit is located at the neck instead of the lower back, as in a conventional PAPR_C_. Consequently, the MFST remained significantly higher compared with PAPR_C_TF_ and PAPR_C_LF_. The MFST was even higher when wearing the full-face PAPR_BR_ than the half-face version, as the full-face mask covered the whole face and provided additional protection from the surrounding cold air. Significant gender differences were observed in MFST_cr_ (while using PAPR_BR_H_) and in minimum local skin temperature (chin, while using PAPR_C_TF_), where males showed on average lower temperatures. However, given the limited sample size, these results cannot be generalised and require validation in a larger population.

When using PAPRs in cold environments, facial skin is exposed to high airflow rates and cold air. Those factors combined may increase the risk of chilblains, frostnip and frostbite (Ducharme and Brajkovic 2005; Brajkovic and Ducharme 2006; Castellani et al. 2006). However, scientific reports on cold injuries specifically associated with PAPR use in cold conditions are lacking. Individuals with respiratory or cardiovascular diseases, older age or poor oxygen absorption may face particular challenges in selecting and using a respirator. While cold air is not typically a causal factor in the onset of respiratory diseases, it can act as a symptom trigger in people with asthma or rhinitis (Koskela 2007). Cold air directed at the face has been reported to increase systolic blood pressure by up to 30 mmHg at −20 °C with wind speeds of 1 to 5 m/s (Stroud 1991). In individuals with preexisting hypertensive cardiovascular disease, this rise in blood pressure further increases cardiovascular stress (Seifert et al. 2013). Even facial cooling alone—such as cooling the forehead and cheek with cold water—has been shown to elevate blood pressure (Schlader et al. 2016). Facial cooling combined with cold air inhalation can induce bronchoconstriction and trigger asthma attacks in both healthy and asthmatic individuals (Koskela and Tukiainen 1995; Zeitoun et al. 2004). Thus, cold exposure and the physiological strain imposed by PAPRs can pose a health risk for people with respiratory and cardiovascular conditions. In contrast, inhalation of cold air (10 °C) has been reported to mitigate increases in deep body temperature and reduce heat strain during exercise in hot environments (Tsuji et al. 2015). Whether a similar effect occurs during heavy physical work in the cold—potentially providing a protective effect against heat strain or, conversely, contributing to excessive cooling of the body—remains to be investigated in the future.

Moisture retention can create discomfort when using FFRs during high workloads. In the present 40-min cold exposure protocol combining exercise and rest, moisture accumulation was significantly greater in FFRd than in FFRe. Most moisture in FFRd (composed of polypropylene and polyester), was retained within the mask material whereas in FFRe (composed of elastomeric compounds), moisture could either escape through the valves or remain in liquid form inside the mask. Wearing a wet FFRd causes discomfort and may increase breathing resistance during prolonged use. Roberge et al. (2010) reported that 0.26 mL of moisture retention over 4 h of FFR use with a valve increased inhalation and exhalation resistance by only 3%, as measured with an automated breathing and metabolic simulator. In a later study, Roberge et al. (2012) found moisture retention of <0.3 g over 2 h of FFR use at a low-to-moderate work rate at room temperature. In contrast, the current study observed much greater moisture retention (>3 g over 40 min for FFR), which may be partly explained by the ambient air temperature of −20 °C. Some of this moisture froze on the mask material, forming a visible layer of frost. If the mask included an oronasal inner mask (as in PAPR_C_TF_), moisture retention was lower compared with the loose-fitting PAPR_C_LF_. The more than 2-fold higher moisture accumulation in PAPR_C_LF_ can be attributed to the large oronasal area covered and the large visor, where cold air and mask material promote condensation, impairing vision. In PAPR_C_TF_, external airflow is restricted to the inner mask, which likely ventilates it effectively and helps expel moisture. Despite the substantial moisture retention observed in this study, visual inspection revealed no freezing of moisture within filters or valves that would have impaired device function or rendered it inoperable.

### Managerial implications

Note that the perception of coldness is highly individual, making it difficult to define strict temperature limits for PAPR use. Nevertheless, occupational cold exposure should never reach levels that induce pain (Gavhed et al. 2000). Employers have a statutory obligation to ensure that the personal protective equipment (PPE) provided is suitable for the specific conditions of use and safe for the user. Cold exposure can cause several harmful health effects, potentially leading to injuries, disabilities and accidents in occupational settings (Tapper et al. 2025). Considering the thermal properties of RPDs in cold environments can help prevent cold-related injuries and reduce the risk of workplace accidents and human error. If respirators are used in temperatures colder than those specified in the user manual (often <−10 °C), employers must conduct a thorough risk assessment. This assessment should consider workers’ thermal comfort, existing respiratory or cardiovascular conditions, the protective capability of the equipment and the level of particulate hazard. Fog-proofing technologies may provide a practical solution for reducing visor fogging and maintaining the field of view in cold conditions (Johnson 2016). The use of PAPR-type RPDs should be limited in duration in cold environments. The implications of respirator use in cold conditions should also be included in employee health checks and workplace surveys.

### Limitations and future studies

This study was conducted in a climate chamber under strictly controlled laboratory conditions, performing tasks representative of light-to-moderate industrial outdoor work. In real occupational settings, environmental factors and worksite characteristics are inherently variable, and work tasks may involve different levels of physical activity, which can influence both RPD use and its thermophysiological effects in the cold. During periods of high physical exertion, elevated respiratory rates and increased facial blood flow may reduce heat loss, potentially raising facial skin temperature in cold conditions. This aspect warrants further investigation in our original research context. It is also important to consider that individual factors, such as physical fitness and body fat composition, may affect facial skin temperatures while wearing RPDs. Furthermore, the duration of measurements in this study was <1 h, whereas a typical workday may last approximately 8 h and include multiple phases during which RPDs must be worn. Work shifts also typically include breaks and may alternate between indoor and outdoor environments, introducing additional variability in thermal exposure. Therefore, future studies should be conducted in real work environments to better understand the complexity of using RPD in cold outdoor work.

Cold conditions may affect the efficacy of RPDs, particularly through moisture accumulation within the mask, filters, or valves and potentially by stiffening the mask materials, which can compromise the tight seal with the facial skin (Johnson 2016). The protective performance of different RPDs should be systematically examined in cold environments using appropriate fit-testing procedures. Moisture accumulation in RPD filters or valves should be measured under cold conditions and, for comparison, at other temperatures to aid the interpretation of the results. Furthermore, future studies should investigate methods for facial warming during PAPR use in cold environments, as this study indicates that such measures have direct implications for worker comfort and safety.

## Conclusion

This study demonstrated the thermal effects of facial skin temperature changes during the use of RPDs in cold conditions (−20 °C). The degree of facial cooling depends on the type of RPD. The face is partially protected from cooling when wearing a nonpowered filtering half-face respirator. In contrast, powered air-purifying respirators accelerate face cooling by convecting cold ambient air across the face. When air flow is controlled by the wearer's breath, facial cooling is less pronounced. Moisture retention within the facepiece is high in cold conditions and can cause discomfort—particularly when using filtering facepiece respirators—and may reduce the field of view when full-face masks are worn. The use of powered air-purifying respirators is not recommended if the facial skin temperature falls below 15 °C. To improve working conditions in the cold while using respirators, especially powered air respirators, methods to prevent excessive facial cooling should be investigated.

## Data Availability

The data underlying this article are not publicly available for ethical and privacy reasons.
